# Are hospitals prepared to support newborn survival? – an evaluation of eight first-referral level hospitals in Kenya*

**DOI:** 10.1111/j.1365-3156.2009.02358.x

**Published:** 2009-10

**Authors:** Charles Opondo, Stephen Ntoburi, John Wagai, Jackline Wafula, Aggrey Wasunna, Fred Were, Annah Wamae, Santau Migiro, Grace Irimu, Mike English

**Affiliations:** 1 KEMRI Centre for Geographic Medicine Research – Coast, and Wellcome Trust Research ProgrammeNairobi, Kenya; 2 Department of Paediatrics and Child Health, College of Health Sciences, University of NairobiNairobi, Kenya; 3 Division of Child and Adolescent Health, Ministry of Public Health and SanitationNairobi, Kenya; 4 Department of Paediatrics, University of OxfordOxford, UK

**Keywords:** neonatal care, hospitals, Kenya, observational study

## Abstract

**Objective:**

To assess the availability of resources that support the provision of basic neonatal care in eight first-referral level (district) hospitals in Kenya.

**Methods:**

We selected two hospitals each from four of Kenya’s eight provinces with the aim of representing the diversity of this part of the health system in Kenya. We created a checklist of 53 indicator items necessary for providing essential basic care to newborns and assessed their availability at each of the eight hospitals by direct observation, and then compared our observations with the opinions of health workers providing care to newborns on recent availability for some items, using a self-administered structured questionnaire.

**Results:**

The hospitals surveyed were often unable to maintain a safe hygienic environment for patients and health care workers; staffing was insufficient and sometimes poorly organised to support the provision of care; some key equipment, laboratory tests, drugs and consumables were not available while patient management guidelines were missing in all sites.

**Conclusion:**

Hospitals appear relatively poorly prepared to fill their proposed role in ensuring newborn survival. More effective interventions are needed to improve them to meet the special needs of this at-risk group.

## Introduction

Over four million newborns die annually worldwide (Lawn *et al.* 2005). Most of these deaths occur in low-income countries of Africa and Asia where the majority of the world’s poor population live (Jamison *et al.* 2006; Lawn *et al.* 2006). Many deaths might be prevented by improved antenatal, intra-partum and early neonatal care (Ayaya *et al.* 2004; Darmstadt *et al.* 2008). Morbidity and mortality from birth asphyxia, for example, may be reduced if effective resuscitation is provided (Bang *et al.* 2005), and this requires only basic equipment and skills (Newton & English 2006; Graham *et al.* 2008). Current strategies to improve neonatal outcomes, therefore, focus on improving such care in both the community and facilities with small hospitals expected to provide effective care to newborns from high risk pregnancies and those referred with serious illness. Although there have been several reports indicating the generally poor state of primary care facilities (Bream *et al.* 2005; Mbonye *et al.* 2007; Armstrong *et al.* 2008) and hospital care in Africa (Nolan *et al.* 2001;English *et al.* 2004; Reyburn *et al.* 2008) there are few data on the specific issue of neonatal care. We were therefore interested to explore the capacity of district hospitals to fulfil their anticipated role in the chain of newborn survival in Kenya.

Comprehensive assessments of the quality of care follow the classical Donabedian approach encompassing measures of structure, process and outcome (Donabedian 1988). Outcomes reflect the change in a person’s or population’s current health status or other valued consequence of care such as length of stay or cost. Outcome measures are of the greatest intrinsic interest, because outcome should, conceptually, aggregate all aspects of care, including those that are difficult to measure, such as patient satisfaction with care received (Mangione-Smith & McGlynn 1998). However, outcomes can be somewhat hard to interpret as they can be affected by more than just the health care received, for example being potentially affected by the nutrition, environment, lifestyle and socio-economic status of populations. Process measures aim to examine what is actually done in giving and receiving care, including adherence to good standards of medical care – clinical history, physical examination, diagnostic tests and therapy, technical competence, evidence of preventive management, co-ordination and continuity of care, and acceptability of care to the recipient. The assumption made here is, given the proper procedures, good health outcomes will tend to result. Process measurements are thus important as the most direct assessments of quality if a defined good standard is available as a benchmark. However, parameters such as technical expertise of health care staff and operator skill that are hard to observe, document or define can be difficult to measure (Mant 2001).

Thus our initial focus was on structural aspects of quality that include assessments of the physical environment, organisation of services, availability of human and material resources and equipment. This seems justified as training aimed at improving knowledge and practise will be largely irrelevant if inadequate structure limits the possibility of improving the process and outcomes of care (McClure *et al.* 2007). Furthermore, lack of basic resources has a negative impact on community perception of quality and utilisation of maternal and child health services (Uzochukwu *et al.* 2004) potentially disrupting the links between community, primary care and hospital that are felt to be key for improving newborn survival.

## Methods

Data reported here were collected as part of baseline surveys for a prospective intervention study that has been described in detail elsewhere (English *et al.* 2008). This study focuses on district hospitals, the apex of the pyramid of primary care, that provide first-referral level services to administrative districts in Kenya with populations typically from 150 000 to 750 000 people.

### Study sites

Eight hospitals were purposefully selected from four of Kenya’s eight provinces, avoiding areas with existing, major hospital management intervention projects, and including those with a minimum of 1000 paediatric admissions and 1200 deliveries annually. Additional criteria described in detail elsewhere (English *et al.* 2009) used in selecting hospitals (illustrated in Table 1) aimed to ensure the hospitals represented the diversity typical of districts and their hospitals in Kenya and to allow future allocation to two relatively balanced groups of four hospitals.

**Table 1:** Characteristics of study sites

Hospital	Malaria transmission setting	Antenatal HIV prevalence High ≥10% Mod = 5–10%	No. of deliveries per year	No. of cots for neonatal admission†	Infant mortality rate, per 1000	Catchment population with income below $2/day (%)	Paediatrician and Medical Officer Interns‡
H1	Intense	High	1750	3	>100	50–70	−
H2	Highland	High	4951	13	∼70	50–70	+
H3	Low	Moderate	7500	9	∼40	∼35	−
H4	Arid	Moderate	2080	4	∼70	50–70	−
H5	Intense	High	1697	6	>100	50–70	−
H6	Arid	Moderate	1799	6	∼70	50–70	−
H7	Highland	High	4235	14	>100	50–70	+
H8	Low	Moderate	3595	11	∼40	∼35	−

† This does not include capacity at other areas such as the paediatric wards where newborns are sometimes admitted.

‡ A cadre of newly generated medical doctors attached to hospitals for 1 year for supervised practical experience.

### Selection of indicators

Our starting point for selecting indicators was a quality of care assessment tool developed by WHO (2002) and adapted to the local context in previous work (English *et al.* 2004). We considered existing indicators belong to one of six logical groupings referred to here as domains (Table 2). These were reviewed for their relevance to the provision of essential care for newborns admitted with one or more of the major threats to survival: birth asphyxia, neonatal sepsis and prematurity, low or very low birth-weight. We retained those with a clear, logical or evidence-based link to patient outcomes for these conditions and where necessary supplemented these with additional indicators reflecting the resource or practise implications implicit in national guidelines for care of these disorders (MOH 2007) largely based on WHO guidance (Irimu *et al.* 2008). We focused on the provision of basic hospital care only.

**Table 2:** Domains and items considered

Domain	Items
Hygiene and safety of facility, staff, caretaker and child	Sinks with soap for hand-washing‡ Cleaning/disinfectant supplies are adequate Sharps are disposed of in a special container preventing accidents Toilets are adequate, clean, and easily accessible The mother has access to running water and to an appropriate space, near the ward, to wash herself and her child Mothers have access to a washing facility, in order to wash hers and her child’s clothes Patients are kept in a clean bed/cot Patients’ beds/cots have mattresses Patients receive clean bed sheets Individual cots to prevent sharing, except for twins‡
Organisation of staff and systems of care	Nurse allocated to full-time duty in the nursery for sick babies Daily round by a medical and/or clinical officer in the nursery Medical care for sick newborns available within first 2 h Sick newborns/young infants are kept separate from healthy babies The most seriously ill children are cared for in a section where they receive closest attention Dose and time are recorded for medications and IV-fluids given Monitoring charts are available, with observations at least four times (six-hourly) daily for critically ill children Management guidelines for common conditions available Dose guidelines for commonly used drugs Routine administration of Vitamin K to newborns (in line with national policy) Routine administration of prophylactic eye drops/ointment to newborns (in line with national policy)
Equipment	Weighing scales for infants Warmer for resuscitation in delivery room‡ Bag-valve-mask device‡ Oxygen source, regulators and tubing‡ Oxygen flow metre‡ Suction equipment Phototherapy equipment‡ Infant warming device in nursery‡
Laboratory services	Measurement of haemoglobin/full haemogram Cross-match and blood bank CSF Microscopy: WBC count Gram stain Measurement of blood glucose Measurement of serum bilirubin Culture of CSF and pleural/peritoneal/joint aspirates/urine
Drugs	Aminophylline – (for treatment of apnoea in line with national policy) (Flu)cloxacillin – injection‡ Glucose 10% (or 50% for preparing 10%) Benzyl Penicillin (Crystapen)‡ Gentamicin‡ Tetracycline eye ointment Vitamin K‡ Phenobarbitone – injection‡ Anti-retroviral drugs/Nevirapine for PMTCT Ceftriaxone/Cefotaxime‡
Consumables, fluids and feeds	Paediatric Cannulae IV fluid giving sets Nasogastric tubes, 6, 8,10 and 12 FG Suction catheters, 8,10 and 12 FG 5% Dextrose solution Full strength Ringers/Hartmann’s & Normal Saline Half strength Darrows with 5% dextrose Newborn formula feed for short term nutritional support

‡ There was a corresponding item in the health worker interview that enabled comparison of this item with the facility observation.

### Data collection

Indicators were included in a standard hospital assessment checklist. Assessments were undertaken by one of three supervisors of survey teams (GI, SN, JW), all clinicians, overseen by the study supervisor (ME) during July–September 2006. All items were scored only as present/absent. Drugs were only deemed available for immediate use if they were found on the ward, thus drugs present only in the hospital pharmacy where access outside routine hours is problematic were classified as unavailable. Equipment was deemed present only if it was functional. Survey teams were trained together for 2 weeks prior to conducting baseline assessments and as part of this training together performed an assessment on a non-study hospital to promote consistency in assessment practises. During baseline surveys, assessors visited each relevant area of the hospital and ascertained the availability of resources and service organisation by direct observation.

As such cross-sectional data may suffer from the well-known problem that resource availability can vary rapidly over time, we also attempted to use health worker opinions on availability to cross-reference our observations where possible. To do this we used a self-administered, structured questionnaire, pre-tested in a non-study hospital, issued to health care workers seeking their opinion on the level of provision of key services and availability of resources. For practical reasons, it was not possible to issue this questionnaire to either all staff or a random selection of workers. Instead it was administered to health workers providing care to newborns during the two-week survey period who had worked in these areas for at least 2 weeks prior to the survey. The tool asked them to indicate, from their recent experience, how often an item was available for use on the last ten occasions when they needed it to provide care to a sick newborn. Thus, they could provide an availability score from 0 to 10 for each item.

### Analysis

Data were double entered and verified using Microsoft Access™ and all analyses were conducted using STATA v9.2 (StataCorp, TX, USA). We have considered each item represented in the facility observation to have equal weight in our analyses. The sum of items present in each domain was determined and the median (and range) of the summed values is used to indicate availability across the eight hospitals for each domain. Simple proportions of all items present within a hospital and across hospitals are also calculated. The median health worker availability scores for each item within each hospital were calculated and median scores <5/10 were taken to indicate absence of an item and scores ≥5/10 presence of an item. Proportionate agreement and chance adjusted agreement (using Cohen’s kappa) between survey supervisors’ assessments and health worker opinions on the presence or absence of items were then calculated.

## Results

### General situation at baseline

Observed availability of items for newborn care varied across the sites. Indicators of availability of drugs, consumables and laboratory services generally scored best. For health worker and patient hygiene and safety, few of the eight hospitals had clean, adequate and accessible toilets or adequate washing facilities for caretakers and their babies (Table 3) but this domain demonstrated the widest within-domain differences between hospitals. One hospital had only 1 of the 10 items surveyed while another had 8. Overall organisation of staff and systems of care were poorest – one hospital failed on all criteria while the best in this domain had only six of the eleven items.

**Table 3:** Availability of items

	Item availability: median per hospital and range across hospitals			
Domain	Min.	Median	Max.	Items available in less than four of the eight hospitals
Hygiene and safety of facility, staff, caretaker and child	1/10	5/10	8/10	Clean, adequate and easily accessible toilets§ The mother has access to running water and to an appropriate space, near the ward, to wash herself and her child§ Patients receive clean bed sheets§ Individual cots to prevent babies other than twins sharing
Organisation of staff and systems of care	0/11	3/11	6/11	Daily round by a medical and/or clinical officer in the nursery Medical care for sick newborns available within 2 h Sick new-borns/young infants are kept separate from healthy babies The most seriously ill children are cared for in a section where they receive close attention Management guidelines for common conditions available§ Dose guidelines for commonly used drug available§ Routine administration of Vitamin K to newborns§
Equipment	2/8	4.5/8	7/8	Warmer for resuscitation in delivery room Oxygen flow metre Infant warming device
Laboratory services	4/6	5/6	6/6	Test for serum bilirubin
Drugs	5/10	7/10	9/10	(Flu)cloxacillin – injection Phenobarbitone injection
Consumables, fluids and feeds	2/8	5.5/8	7/8	Newborn formula feed for short term nutritional support§

§ Available in 0 or 1 site only.

The hospital selection criteria resulted in the two larger of the eight sites having a consultant paediatrician which is generally very uncommon in Kenyan district hospitals. Despite this in only one of eight hospitals was there a daily, week-day ward round by a clinical or medical officer of the nursery where sick newborns were cared for. There were no daily, week-day nursery ward rounds by the paediatricians. More worryingly, in six of eight hospitals there was no nurse specifically allocated to duty in the newborn nursery, in most hospitals nursery cover was provided by nurses also having to offer full-time service to the delivery room or post-natal ward. In six of eight hospitals nurses were expected, because of the absence of clinicians, to provide acute medical care in addition to nursing care in the first 2 h (and often considerably longer) after delivery or admission of a sick newborn. Such care could include resuscitation and initiating treatment with parenteral antibiotics, anti-convulsants, phototherapy, intravenous fluids, or assisted feeding while a formal clinician’s assessment was awaited.

Unfortunately guidelines for management of common, life-threatening neonatal conditions or for prescribing drug doses, fluids and feeds were rarely available with both nurses and clinicians ‘doing their best’. The difficulties in offering appropriate care are also illustrated by the inability of half of the hospitals to offer phototherapy of any kind while stationery and personnel for monitoring the clinical condition, intravenous fluid therapy or feeding for even the sickest babies were usually lacking. Vitamin K was routinely administered to newborns in only one hospital although available in seven. Tetracycline eye ointment prophylaxis was routine in three hospitals but available in five (Table 4).

**Table 4:** Recommended essential care practises and standards

Essential care practises and standards	Number of sites (out of eight) in which found/possible
Management guidelines for common conditions available	0
Dose guidelines for commonly used drugs	0
Administration of Vitamin K to newborns as a routine hospital policy	1
Feeding volume guidelines available	3
Sick babies close to the nurses’ station for careful monitoring	3
Newborns can get phototherapy	4
Basic observations of the sickest babies are taken at least six-hourly	4
Administration of prophylactic eye drops/ointment to newborns as a routine hospital policy	4
Fluids, feeds and drugs are monitored in the sickest few children	5
Babies can be kept warm	7

**Figure 1:**
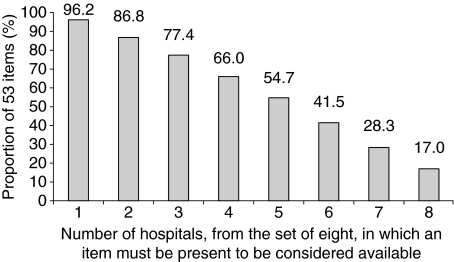
Availability of 53 items as criteria for availability are varied from presence in one of eight to all eight of the hospitals within the set.

Examining the proportion of the 53 items that were present in the eight hospitals (Figure 1) indicates that only 17.0% (nine items) were found in all eight hospitals, and 3.8% (two items – management guidelines for common conditions and dose guidelines for commonly used drugs) were missing in all of the hospitals.

### Comparing the facility observation with health worker-reported availability

**Figure 2:**
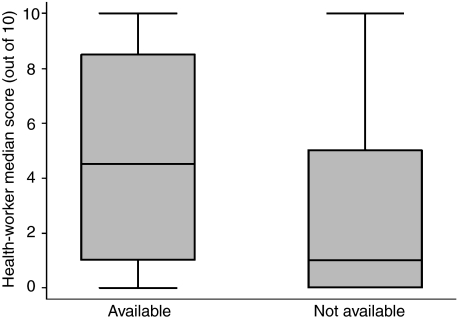
Boxplot of hospital specific, median health worker availability scores for each of 14 items categorised as available/unavailable in the same site by direct observation of the survey supervisor (central line = median of median scores, shaded box = inter-quartile range of median scores, ‘whisker’ = 95% range of median scores).

It was planned to ask the opinions of health workers about the availability of 17 of the 53 items assessed by observation, resulting, across the eight hospitals, in 136 potential comparisons of observation and the median of health workers’ opinions. However, during the initial surveys it became clear for three of these 17 items that health workers’ interpretation of the self-administered questionnaire would preclude sensible comparison with survey workers’ observations. Responses were available from 3 to 20 health workers per site, the smallest number in the site with nurses allocated specifically to the nursery. Comparing the 14 items across the eight hospitals (112 comparisons) the findings of the two tools were similar in 72 comparisons (62.5%, kappa = 0.26, *P* = 0.003); neither tool systematically indicated a higher degree of availability. Exploring agreement between observation and opinion for each survey team supervisor individually demonstrated similar-to-overall agreement (64.3%, *n* = 39, kappa = 0.28, *P* = 0.03; and 66.7%, *n* = 30, kappa = 0.35, *P* = 0.01) for two supervisors while for the third agreement may have been less good although lack of data (53.6%, *n* = 12, kappa = 0.011, *P* = 0.5) limit truly meaningful interpretation. Comparison of the medians of 112 health worker scores for the 14 items defined by the observers as present (*n* = 61)/absent (*n* = 51) across the eight hospitals demonstrates that scores were generally low (Figure 2).

## Discussion

In this study we used observations made by trained survey staff to examine fundamental structural components required to provide care for sick newborns with the most common causes of newborn mortality in line with WHO and national guidelines in eight Kenyan government district hospitals. These hospitals represent a relatively small, non-random sample of all Kenyan hospitals and therefore our results should not be used in any specific, quantitative sense to describe the situation in Kenyan hospitals generally. However, we feel our results do illustrate some of the likely areas in which structural aspects of care for newborns are deficient, a view supported by non-survey visits by some of the authors to many other hospitals in Kenya. Our findings suggest that important, structural components for providing newborn care were often unavailable at the time of baseline surveys in the eight sites (Table 3). Specific problem areas were noted; for example with regard to infection prevention where inability to separate out-born infants from those born within the hospital, lack of appropriate cleaning materials on the wards and inadequate toilets and washing facilities for the mothers were common. Oxygen supply and delivery systems, resuscitaires and bag-valve-mask devices are vital equipment in facilities expected to provide emergency obstetric and newborn care, these too were often unavailable although some of these resource shortfalls have since been tackled. Such physical problems were commonly linked to very limited availability of guidelines for care and inadequacies in systems or organisation of care. For example, no hospital had clinical management guidelines for common causes of serious illness in newborns; in most hospitals no clinician provided routine review of sick newborns and although available in seven hospitals, no hospital was adhering to the government policy to provide routine Vitamin K at birth, perhaps because of the formulation of Vitamin K supplied.

Cross-sectional observations such as these may be criticised for providing an estimate of point-prevalence in availability only, arguably a problematic measure when trying to assess a dynamic, working hospital environment. It is therefore useful to examine the context of care from more than one perspective and compare the findings. We attempted to examine the credibility of our findings by using health workers’ opinions of availability for some items as an estimate of recent period-prevalence. An alternative or complimentary approach might be to seek caretaker opinions on availability of resources even though their lack of technical knowledge might make this problematic. Although measuring somewhat different aspects of availability there was some agreement between the survey observations and health worker opinions, providing some reassurance that our findings are reasonably indicative of the reality and in general health worker reports of availability, on a scale of 0–10, were relatively low (Figure 2). However, there were also sometimes discrepancies for which several explanations other than the different periods of measurement are possible. Thus, there may be differences in interpretation of ‘availability’. For example in one case the survey workers observed a drug to be available but health workers reported the same drug as not being available because mothers had to pay for the drug before it was provided. It is also possible that the relatively small (especially in some sites) and convenience sample of 81 respondents lead to chance misclassification. Different assessment practises of the team supervisors, as suggested by the (non-significant) lower agreement between approaches in one of the three teams, and reluctance of health workers to give high scores could also contribute to apparent disagreement between data obtained by observation and health worker response.

Availability of essential items for provision of care is a widely used indicator of quality of care (Litvack & Bodart 1993; Gilson *et al.* 1995; Kamat 1995). It is based on the assumption that given the proper resources and organisational structure, health care workers are enabled to provide good quality services; conversely, poor organisation, resources and infrastructure are likely to be associated with poor quality of care. The latter concern is of especial relevance to low-income settings where inadequate resources are often reported (UNDP 2007) and where, in our experience, there are few local ‘champions’ advocating for the needs of newborns.

## Conclusion

Even reasonably large rural hospitals (including those with paediatricians) may be poorly prepared to offer key services to sick newborns. To prevent or reduce the four million newborn deaths it will be important to ensure that all parts of the ‘chain of survival’ are adequate. Simple, cost-effective and sustainable interventions will be required to improve hospital systems to cater for the special needs of newborns, especially if community or primary care level interventions result in increasing referral rates. Such interventions will need to move well beyond the tradition of delivering training courses alone.

## References

[b1] Armstrong SJR, Mrisho M, Manzi F (2008). Health and survival of young children in southern Tanzania. BMC Public Health.

[b2] Ayaya SO, Esamai FO, Rotich J, Liechty E (2004). Perinatal mortality in the special care nursery of Moi Teaching and Referral Hospital, Eldoret, Kenya. East African Medical Journal.

[b3] Bang AT, Bang RA, Baitule SB, Reddy HM, Deshmukh MD (2005). Management of birth asphyxia in home deliveries in rural Gadchiroli: the effect of two types of birth attendants and of resuscitating with mouth-to-mouth, tube-mask or bag-mask. Journal of Perinatology.

[b4] Bream KD, Gennaro S, Kafulafula U, Mbweza E, Hehir D (2005). Barriers and facilitators for newborn resuscitation in Malawi, Africa. African Journal of Midwifery and Women’s Health.

[b5] Darmstadt GL, Walker N, Lawn JE, Butta ZA, Haws RA, Cousens S (2008). Saving newborn lives in Asia and Africa: cost and impact of phased scale-up of interventions within the continuum of care. Health Policy and Planning.

[b6] Donabedian A (1988). The quality of care: how can it be assessed?. JAMA.

[b7] English M, Esamai F, Wasunna A (2004). Assessment of inpatient paediatric care in first referral-level hospitals in 13 districts in Kenya. The Lancet.

[b8] English M, Irimu G, Wamae A (2008). Health systems research in a low-income country: easier said than done. Archives of Disease in Childhood.

[b9] English M, Ntoburi S, Wagai J (2009). An intervention to improve paediatric and newborn care in Kenyan district hospitals: understanding the context. Implementation Science.

[b10] Gilson L, Magomi M, Mkangaa E (1995). The structural quality of Tanzanian primary health facilities. Bulletin of the World Health Organization.

[b11] Graham SM, English M, Hazir T, Enarson P, Duke T (2008). Challenges to improving case management of childhood pneumonia at health facilities in resource limited settings. Bulletin of the World Health Organization.

[b12] Irimu G, Wamae A, Wasunna A (2008). Developing and introducing evidence-based clinical practice guidelines for serious illnesses in Kenya. Archives of Disease in Childhood.

[b13] Jamison DT, Shadis-Salles SA, Jamison J, Lawn JE, Zupan J, Lopez AD, Mathers CD, Ezzati M, Jamison DT, Murray CJL (2006). Incorporating deaths near the time of birth into estimates of the global burden of disease. Global Burden of Disease and Risk Factors.

[b14] Kamat VR (1995). Reconsidering the popularity of primary healthcare in India: a case study from rural Maharashtra. Social Science and Medicine.

[b15] Lawn JE, Cousens S, Zupan J, Lancet Neonatal Survivial Steering Team (2005). 4 Million neonatal deaths: when? Where? Why?. The Lancet.

[b16] Lawn JE, Zupan J, Knippenberg R, Begkoyian G, Jamison DT, Breman JG, Measham AR (2006). Newborn survival. Disease Control Priorities in Developing Countries.

[b17] Litvack JI, Bodart C (1993). User fees plus quality equals improved access to healthcare: results of a field experiment in Cameroon. Social Science and Medicine.

[b18] Mangione-Smith R, McGlynn EA (1998). Assessing the quality of healthcare provided to children. Health Services Research.

[b19] Mant J (2001). Process versus outcome indicators in the assessment of quality of health. International Journal of Quality of Health Care.

[b20] Mbonye AK, Mutambazi MG, Asimbwe JB (2007). Declining maternal mortality ratio in Uganda: priority interventions to achieve the Millennium development goals. International Journal of Gynaecology and Obstetrics.

[b21] McClure EM, Carlo WA, Wright LL (2007). Evaluation of the educational impact of the WHO essential newborn care course in Zambia. Acta Paediatrica.

[b22] Ministry of Health (2007). Basic Paediatric Protocols.

[b23] Newton O, English M (2006). Newborn resuscitation: defining best practice for low-income settings. Transactions of the Royal Society of Tropical Medicine and Hygiene.

[b24] Nolan T, Angos P, Cunha AJLA (2001). Quality of hospital care for seriously ill children in less developed countries. The Lancet.

[b25] Reyburn H, Mwakasungula E, Chonya S (2008). Clinical assessment and treatment in paediatric wards in the north-east of the United Republic of Tanzania. Bulletin of the World Health Organization.

[b26] United Nations Development Programme (2007). Human Development Report 2007/2008.

[b27] Uzochukwu BS, Onwujekwe OE, Akpala CO (2004). Community satisfaction with quality of maternal and child health services in southeast Nigeria. East African Medical Journal.

[b28] World Health Organization Improving quality of paediatric care – assessment tools: pretoria 2001. http://www.who.int/child-adolescent-health/publications/IMCI/HFS.htm.

